# Impact of digital communications on project efficiency through ease of use and top management support

**DOI:** 10.1016/j.heliyon.2023.e17941

**Published:** 2023-07-05

**Authors:** Kainat Afridi, Jamshid Ali Turi, Barirah Zaufishan, Joanna Rosak-Szyrocka

**Affiliations:** a Public Information & HR Professional, United Nations Information Centre, Islamabad, Pakistan; b University of Tabuk, Tabuk, Kingdom of Saudi Arabia; c Management Studies, Bahria University, Islamabad, Pakistan; d Department of Production Engineering and Safety, Faculty of Management, Czestochowa University of Technology, Poland

**Keywords:** Project performance, Digital communication tools, Top management support, Project management, Telecommuting, Work from home, Ease of use

## Abstract

The pandemic has made the world witness an overnight transition from traditional to digital space owning to a lockdown, affecting everyday life. During the lockdown, the business community and people switched to online communication tools for survival. The behaviour towards adopting online communication tools during the pandemic has opened a new direction for researchers to study. This study aims to understand how during the covid-19 lockdown, digital communication tools affect project efficiency. It also explored to what extent project performance (PP) has been affected by digital communication during the lockdown. The study has used a cross-sectional quantitative-based approach. Moreover, a convenience sampling technique was adopted to collect. The target respondents are the project staff and employees of humanitarian organisations comprising international non-profit organisations (INGOs), UN agencies, and local non-profit organisations (NGOs) based in Pakistan. The survey was shared with 400 employees, and only 302 respondents responded. The results show that the user behaviour towards adopting digital communication tools is high; digital communication tools have an impact on project performance, while ease of use significantly moderates the relationship between digital communications tools and project performance compared to top management support. The research helps study employees’ struggles in adopting change and advising organisations to devise practical digital communication strategies and business continuity plans to cope with crises.

## 1 Introduction

With Covid-19 being declared a pandemic in February 2020, the effects were universal and unprecedented in scale. As the world went into lockdown, this increased dependence on digital communication mediums resulted in a gush in internet use. In April 2020, an increase of 7% in internet users compared to April 2019 was reported [1]. The report indicates that digital media use witnessed a stark rise, e.g. digital conferences, virtual meetings, online teaching, and summits, reflecting the switch to online communication modes. The pandemic sped up the transformation from offices to online communication modes. Spontaneous changes in operations emerged with restrictions imposed by local and federal governments during the lockdown. It resulted in downsizing, restraining operations and projects, and freezing the funds. To survive this crucial lockdown phase, humanitarian organisations/projects in Pakistan shifted their sails and revised their strategy by adopting remote working to survive the lockdown phase.

Communication is of the utmost importance in our everyday life, personal or professional. Communication originated from the Greek word ‘Communicate,’ which means ‘to Share’. It is a process where participants interact to share meaning and understanding [2]. In today's globalised world, its indispensability is evident from the fact that it has become vital for the success and survival of both humans and the organisation—organisations practice organisational and corporate communication. The former is the internal flow of communication, and the latter manages the relationship with external stakeholders [3]. Many international and national organisations like United Nations (UN) have an added program - communication for development [4]. This approach uses a blend of communications tools and strategies to engage the community and emphasises two-way communication to allow a discussion for positive behaviour/social change in the humanitarian context [5].

In the past, job holders voluntarily did remote work, particularly consultancies that were outsourced to be home-based or task-based. Currently, the organisation adopted alternate working arrangements due to the pandemic. The covid-19 pandemic differs from the previous one – the Spanish Flu 1918–1919 during World War [6]. Previously, human-made or natural disasters would cease the usual routine of human life and significantly have economic consequences on industries and labour. Covid-19 directly affected the country's GDP by 6% [7,8]. Nevertheless, with the novel SARS-CoV-2 virus, the situation is different from the last pandemic.

Organisations turned to telecommute, increasing the demand for information and communications technology to replace the traditional workplace [9,10]. During the pandemic, adopting telecommuting has been exhausting and challenging for employees and humanitarian organisations. However, employees faced impediments in work and communication-related hurdles in work speed [11]. The alternate work modalities require more than average attention; people work without taking breaks due to the fear of not meeting productivity goals, which may result in job loss [12]. It also puts unintended pressure on employees to be well-versed in using the latest digital communication tools for better performance remotely. Thus telecommuting blurred the lines between personal and professional life, causing draining effects on employees, fatigue, anxiety, burnout, and depression [12,13].

To organisations whose success was built in the pre-digital economy period, digital tools are an opportunity and threat to their existence. Most successful organisations are big old companies; therefore, adopting digital transformation is cumbersome [[14], [15], [16]]. Additionally, organisations need cultural values to master digitalisation [17] -. However, digital transformation during a crisis was different. The organisations may need to put all their formalities to the back, and the only consensus they need is to take the risk of using digital tools [18].

During the Pandemic, a study on psychologists’ acceptability of using new digital information and communication tools for counseling was conducted [19]. However, the study lacks how and which digital tools psychologists were asked to use by their organisations. Was it social media channels, already available digital tools like Teams, zoom, and Skype, or customised applications meeting the needs of an organisation?

The pandemic has affected the usual business of organisations and an ordinary person's life while coping with the lockdown and related restrictions. During this time, most people who rely on face-to-face communication and prefer to build a connection through meeting in person have faced implications. The stay-at-home and self-distancing made it more difficult, as less tech-savvy people need to familiarise themselves with the tools and their usage. At the same time, the older population and people with lower internet skills or limited availability have reduced the use of digital tools during a pandemic to stay connected. It may lead to isolation and depression at a time when the only way to remain connected is through digital mediums [20]. However, on the professional front, we have similar people with professions or personalities, the less tech-savvy, who prefer to work closely with their teams. These people have a good command of communication skills regarding in-person conversation, but covid-19 lockdown brought new challenges for them. They need to upgrade their skills and understanding of new Information and communication tools and adjust to the new working mode-working from home (telecommuting). The research lacks to shed light on how professionals, despite the challenges of telecommuting and using new ICT tools, could achieve or are on the road to achieving performance goals, how they could overcome the new normal challenges while working in front of an LED the whole day. Were they satisfied with the step taken by their management to handle the remote working?

Project performance during the pandemic did affect by human resource knowledge, ease of use of the technology of communication, and the support provided by the top management of the organisations/projects. Adopting the virtual workplace has been hard on employees due to the absence of in-person touch, but it brought diversity to the workforce with geographic dispersion. Technology innovation may innovate digital tools, but their success depends on the end-users perceived ease of use. The comfortability of using a digital tool and the support provided for navigating through it significantly affect the trait of acceptability of human behavior [21].

Project performance literature is found to be silent on communication and project performance. Therefore [18], identify to study the impact of digital communications on employees’ personal and professional lives during disasters. Similarly [19], and [20] asked to assess the effect of digital communication on project performance during the pandemic of Covid-19, when the workers were working remotely. Therefore, this study aims to cover the literature gap mentioned above to assess the drastic effects of digital communications and the gaps in the project and performance goals.

Based on the objectives, the study investigated to answer: “What is the relationship between digital communication and project performance with the moderating effect of the ease of use of communication technology and top management support? Therefore, the main queries this research answered were “How have digital communications affected the project performance during the covid-19 pandemic? and “How has the top management moderated the use of digital communications and project performance?”

## 2 Literature review

### 2.1 Digital communications (DC)

In the past decade, communication in crisis was reactive; once the disaster happened, communication would follow. This put employers in a challenging situation during a pandemic when the working environment shifted to telecommuting. For organisations, telecommuting may decrease costs and increase productivity (PEARCE, 2009), but it is an increase in employee responsibilities or downsizing. The humanitarian organisation projects use different digital communication tools, from emails to social media, that are effective during covid19 lockdown [[22], [23], [24]]. The purpose of digital communications tools is to connect socially and professionally during a lockdown, considering that 13% of the jobs in Pakistan can be performed from home [25]. Apart from leadership, effective and transparent communication is a crucial element identified while working on the projects [26]. However, digital transformation could mean a higher or lower presence in the digital space [27].

Effective and transparent digital communication is possible if people are digitally literate to understand and communicate. In Pakistan, the literacy rate is 60%, comprising 71% of men and 69% of women. Internet facility is available to 34%, while 51% are in urban and 34% are in rural areas [28]. With limited internet facilities and disruption due to the increased load on internet service providers, even the best digital tools would be of little use. The digital tools only help to transmit the message, but a correct channel selection with the right message would allow the project management to achieve project goals and stimulate resilience [29]. Since the Pandemic, United Nations World Health Organisation has continuously used social media channels to keep the public aware of the evolving risks and measures to adopt [30]. Social media channels are easy to understand and do not require extraordinary skills to operate.

Digital communication in crisis is challenging and critical as one is unsure of the other side. Similarly, on October 8, 2005, Pakistan's northern areas were hit by a massive earthquake [31]. Click or tap here to enter text.in the initial hours, there was no news until the military, due to losing connection with their base, reached out to them. The information about the massive destruction of infrastructure and human loss broke out [32]. So, no matter how well prepared and robust strategies projects/organisations have, in the end, communication in disasters is difficult as the destruction/viral infectious diseases could wipe out the whole community. The only way to reach them is to go on the ground. As a result of the pandemic, workplace digitalisation has expedited, changing the projects' spectrum of work and requirements [33].

### 2.2 Ease of use of digital tools (EU)

With the influx of information and technological advancement, people are prone to use different mediums for efficiently connecting and being productive. Every day, new technologies like augmented reality (AR), artificial intelligence (AI), virtual reality, and machine learning are helping to create and innovate new technological tools for sharing, curation, and content marketing. Artificial intelligence (Al) supports language and content generation, customer-oriented chatbots, and human voice search [[34], [35], [36], [37]].

Covid-19 has accelerated the digitalisation process; many customer-oriented businesses have moved to digital platforms, and so have the customers. The profit of digital businesses has soared to an all-time high, like Alibaba-the mogul of the Chinese digital industry has seen a 34% increase in revenue until June 2020 [38]. Women in UAE use Facebook and WhatsApp as communication tools which have yielded results in revenue and growth [39] . Similarly, WebEx, Quill, Trello, and Dischord are used for meetings, data transformation, project management, and instant messaging [37,40].

Davis [41] defines the perceived ease of using technology as “the degree by which a person believes that using technology would be effort-free”). Accordingly, the comfort of using a digital tool and the support provided for navigating through it significantly affect the trait of acceptability of human behavior [21]. User acceptance highly depends on the system's usefulness to the users [42]. A study concludes that ease of use mediates the perceived usefulness of technology and an individual's self-confidence [43]. The organisations can improve the perceived ease of use of digital communications tools users face that they opt for using during the initial design. The argument that low user acceptance is due to the user's high efforts contrasts with the belief that the same user may put more effort if the system would help meet the desired outcome [44].

Different technological mediums help employees and users achieve personal and professional goals [45] . If the technology adopted has a rugged user face and staff face challenges in using it, the tool would be unimportant to individual and team/Organisational performance. It may cost the organisation financially, but it will not contribute to achieving the goals on the ground; the performance may get affected.

### 2.3 Project performance (PP)

Performance management was traced and mentioned in 1813 when a General in the US army submitted his men's evaluations to the War Department. A General's performance rating of his men can be viewed as the start of individual performance management [46]. Performance is a multifaceted construct depending on various factors for its measurement [47]. Previously management of performance was part of Human Resource Management; however, the evolution of the different fields and integrated work of various departments has taken the performance in the organisation context [48]. Micheal Armstrong has defined Performance management as an ordered process of integrating teams' and individuals' performance. In a traditional setup, performance management is measured by the annual performance reviewers (individual level- HR); however, it has different parameters within the organisation-centric aspect. The organisation's performance management is a part of the project management plan helping the projects meet the goals set in scope [49].

Projects set their goals according to the organisational mission and measure their performance by triple constraint - time, cost, and scope. At the same time, quality is the fourth one, but it is more associated with the scope [50] (−). In the past, time, cost, and quality were termed the iron triangle and preferred criteria by the practitioner for project performance measuring [51]. The measurement criteria of the iron triangle were later called PP [52]. It informs management if the work is behind, at, or ahead of the plans of schedule, cost and quality. Accordingly, the management may take the necessary steps to move as per plan or navigate through alternative methods to meet the project's deadlines and set scope.

Project performance (PP) is unclear and interpreted by the practitioners differently, except where the word itself is more defined [53]. However, PP in literature has been researched with different variables, but in general, efficiency has been termed as a short-term benefit [54]. Over time, multiple elements like stakeholders or sponsors are added compared to the conventional measurement of efficiency in the projects [55]. The details of PP evolve in the environment they perform [56].

In a crisis, performance is not affected by an individual project but also by the parent organisations. It's an essential criterion for project success [57,58]. Project performance does not end if the project completes within the provided cost, schedule, and quality, but it also includes satisfying the customer. Also, completing a project impacts other projects' performance within the Organization [56].

Research shows that communication also plays a salient role in project success [59]. Communication has been continuously evolving with technological advancements. Social media's proliferation has made a simple message evenly essential and critical when coming from the official handle of a company or an organisation. In the current digitalised time, digital communications have affected performance management, which is why Organisations are moving to cloud-based networks and working remotely. The rise in cloud spending is 37% as the Pandemic accelerates the virtual workplace [60,61]. Moving to a virtual workplace project needs the latest digital communication and performance measures tools, as traditional performance management models are redundant, especially during a pandemic [62]. Lacking good communication policies and strategies has often resulted in miscommunication between the stakeholders and management, leading to project failure.

### 2.4 Top management support (TMS)

Successfully implementing decisions, adaptability to change, or alternative working mode during a crisis depends on management support. It is referred to as project leadership, rarely mentioned in the literature on an individual level [63]. It may include an individual or a team, Zirger (90 CE) has termed it top management support – a pulling force to unite the project's different functions.

Management support is an internal factor crucial for achieving impactful project performance [64]. Literature on the TMS is not as rich, but it is a critical factor and extends support to the projects through different behavioural support profiles; resources, communication, power, and structural arrangement [65] . These profiles are taken under this study. It explained the kind of support shown by the top management during the pandemic towards the projects. However, TMS showed flexibility while adapting to the disrupted and unanticipated global crisis that changed and affected everyday life.

Providing the resources during the pandemic is of utter importance. Traditionally, the typical business continuity plans and related financial resources allocated for a pandemic would be low in the regular budget planning. Management is to support the project by providing necessary funds and other resources [66]. Keeping in view, the probability of such events is once in a lifetime. It decreases physical resources’ importance, i.e., materials like office space, and financial and human resource requirements could be low too. This may imply to the Top management to reduce the resources or downsize and restrain the financial sources.

In a crisis, communication is essential to support. It could be formal/informal with the project team, top management team, and other organisational staff [67] [68]. (et al., 2008) has considered encouraging the groups and addressing the problems, stimulating cross-functional cooperation through effective communication. During the Pandemic, communication frequency and information are vital, resulting in increased responsibilities or capacity building of the project team [69,70]. Covid-19 put the management in the spotlight and pressured them to take measures for the project staff's care, have a conversation and build relations [70].

The management team's support for the project with resources is evolving and earned through the project teams' constant efforts. They are the people who can create a supportive environment built on trust to achieve their performance goals [71]. TMS is the balance of power hierarchy, empowering the relevant people using authority and structural arrangement in projects playing a steering role for success in the disrupted global peace.

The hierarchical structure and power/authority distribution play a vital role in steering the projects during a challenging situation. Management support is constructive if the power is used to execute and implement change within the evolving context, including addressing the power struggle among the project team. Issues that hinder progress need to be identified and addressed promptly, using political capital to avoid loss [72]. Another essential factor that is overlooked is TM's expertise. Skills and ample knowledge of the developing situation and implications of the proposed strategy are pertinent to project success. TM's expertise means supporting the teams and projects by providing guidance and knowledge [65,73] -).

A crisis is the mother of invention. A pandemic does not seem to contain calls for innovative approaches; organisations develop innovative strategies to address projects' current and future problems beforehand [74]. The public's eye on top management in parallel to the pandemic-affiliated challenges has forced them to take a people-oriented approach considering economic and business realities. The actions have set the tone for others to understand and follow during the crisis-). It was not easy, yet it was the leaders' Kairos to achieve their project goals.

### 2.5 Theoretical support to the study

The study of human behavior using technology depends on its comfortability and familiarity. The technology is of no use if it is of no use to the user; however, the system's usefulness aside, the ease of use and its useability could affect human behavior. The ease of using the technology affects individual performance in their workplace and personal relation while remaining confined in homes during a lockdown.

The Technology Accepted Methodology (TAM) was developed in 1989 to measure the technology's perceived usefulness and ease of use. The scale thrived shows the empirical relationship between user behaviour, usage intention, and ease of use [41]. User intention is the decision to behave in a particular way. TAM can increase predicting usage and user behaviour if organisational and social factors could be embedded into the model [75]. TAM has been used in different fields like medicine and has helped explain and predict user behaviour using technological tools after their implementation.

Digital communication tools that the employees use may differ in their usage depending on how user-friendly interface the tools hold. The technology tools’ usability depends on their usefulness to the users, as the ease of use will support and make the performance achievable.

### 2.6 Research hypothesis

- Authors explicitly discusses communication's vitality as necessary as breathing [76,77]. In business, communication is a coordinating factor between the departments and people/stakeholders [78]. During a pandemic, critical organisational decisions in thriving and challenging conditions depend on the leadership - top management understanding the situation. Amid a pandemic, organisations adopted new digital tools for working offshore; nevertheless, successfully implementing decisions, adaptability to change, or telecommuting during a crisis/emergency depends on management support. It is a critical factor in being receptive to the change brought by the Pandemic. Management supported adopting digital tools for the survival of their business, but digital tools; selection also depended on their ease of use to achieve project performance. The organisaion's performance management is a part of the project management plan as monitoring and controlling project work. Based on the above premises, theories, and findings, the study hypothesis that:

H1 Digital Communication tools significantly impact project performance

H2 Top management moderates the relationship between digital communication tools and project performance.

H3 Ease of use significantly moderates the relationship between digital communication tools

**Fig. 1:**
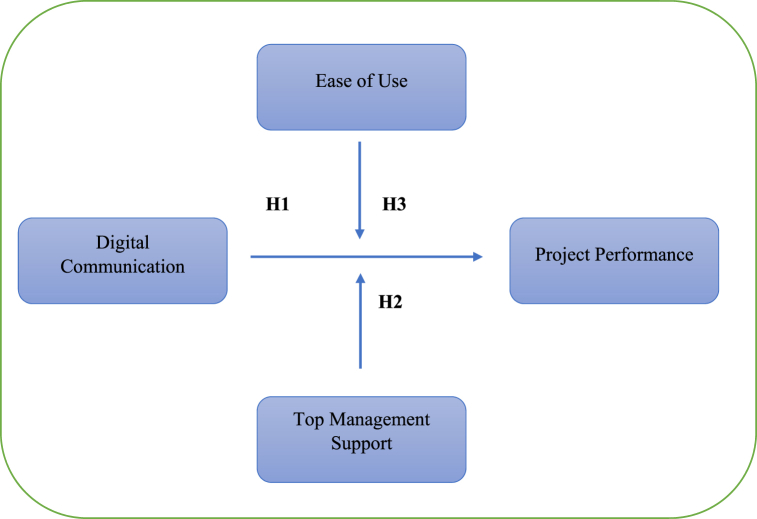
Research model (developed for study).

The study came with the research model based on the cited literature and hypothesised relationship depicted in Fig. 1.

## 3 Methodology

The research has focused on the humanitarian sector and its project to lay out the impact of the independent variable – Digital communications on the dependent variable – Project efficiency/performance. There are two moderators Top management support and ease of use of communication tools.

### 3.1 Design and participants

Zikmund (2003) [79] defined research design as an index of the researcher's manual working on the research. It is also called a strategy of action. The research is cross-sectional in nature [80], allowing quantitative data collection. The selection of cross-sectional design is data for all the variables collected once without influencing them [81]. The population identified as employees working for humanitarian organisations, i.e. the United Nations (UN) agencies, International Non-Governmental Agencies (INGOs), and Local Non-Governmental Agencies (Local NGOs) in Islamabad. Due to the prevailing pandemic conditions, the field offices have a time paucity and internet connectivity issues. However, the strength of staff working for 94 registered INGOs and Local Non-Governmental agencies is unavailable [82]. This makes the population size unknown.

### 3.2 Sampling technique

Since no accurate data is available on the humanitarian sector workforce, the study adopted the convenience sampling technique, a non-probability method. The study participants are selected based on their willingness and availability [83]. The sampling frame is difficult to identify. So for unknown population size, the Cochran formula will be used [84]. Researchers find the sample of unknown population size using Cochran's formula $n_{0}=\frac{z^{2}\times p\left(1-p\right)}{e^{2}}$, Whereas, n_0 – a sample size that was estimated, z^2 - a selected critical value of the desired level of confidence/risk, p - the estimated proportion/maximum variability of the population and e − the desired level of precision/margin error. The value used for estimating the sample size is n_0 - Unknown (?), z^2–95% confidence level (the value of (1- ɑ) in Standard Normal Distribution z-table, which is 1.96 for 95%), p - 50% variability of the population (which is maximum) and e − 5% margin of error. Putting the value in given formula n_0=((1.96)^2 × 0.5 (1–0.5))/(0.05)^2 = 384.16

### 3.3 Unit of analysis & instrument selection

The study subject is project employees of the humanitarian sector, such as project managers, project team leads, project teams, and project consultants – Individuals working in the humanitarian sector projects in Islamabad, Pakistan. Moreover, the scale was selected from the previous studies, attached in Appendix-A, and adapted to the study context.

### 3.4 Data collection and analysis procedures

Data is collected through a survey. The survey questionnaire is divided into five sections. The first explains the subject details and their difficulties using digital communication tools. The rest four sections have demonstrated the variables – digital communications, ease of use of the tools and shed light on project efficiency and the support provided by the top management during a pandemic are given in the appendix.

All of the sections are part of a single questionnaire. The questionnaire's measuring scale is as per the approved TAM model survey questionnaire. However, the TAM survey has seven Likert scales to measure the responses, while this study has used only five Likert scales. Initially, the total number of questions/items in the survey was 63. Digital communication consists of 11 items on a 5 Likert scale ranging from 1 (strongly agree) to 5 (Strongly disagree). The ease of use with 11 items, top management support with 13 items and project efficiency/performance with 13 items followed the digital communication's scale anchor. The data cleaning reduced the items of all the variables; digital communication was left with six items, ease of use with five, eight for top management and project performance with six items.

For data collection, the survey link was widely shared on LinkedIn, emails, and WhatsApp among 400 people from humanitarian organisations. The response time was slow, and it took more than two months to receive 302 responses. The people were contacted through references, so the response received was all relevant and complete – no missing values. The 302 responses were entirely considered.

Once the data was collected and exported to SPSS, the data was transformed from strings to numeric, and the analysis process was initiated with confirmatory factor analysis, followed by reliability, descriptives, correlation, and regression analysis. During the confirmatory factor analysis, the data was cleaned for further analysis. The study tests the relationship between the independent variable – digital communications and the dependent variable – project performance/efficiency; reliability, regression, and correlation are used as data analysis techniques to extract valuable information from the chosen variables.

### 3.5 Ethical consideration

At the front of the survey, information about the respondent's rights to privacy and data confidentiality was mentioned. Their participation is voluntary, and their information won't be shared with a third party. The participants were not harmed or forced to participate, and the correspondence related to the research has been kept transparent and honest. In addition, the data is collected anonymously, and email addresses which the researcher is required to understand if the data entry is coming from the target respondents were removed after gathering data to guarantee the information confidentiality. Data is presented in its original form; no modifications are done to change its reflection pattern.

## 4 Results

Data analysis includes the Confirmatory factor analysis (CFA) and regression analysis. A reliability and validity test was also conducted to ensure the data was valid and reliable, leading to hypothesis testing. The sample size was 384. However, we were able to achieve 302 survey responses within the time frame of two months.

### 4.1 Respondents’ demographic characteristics

Table 1 shows that 32.8% of females and 62.9% of males participated in the survey. The results reflect that 65.2% of respondents have higher education holding master's degrees. At the same time, 29.1% have 11–15 years of experience working. As a part of a study conducted during a pandemic, one of the crucial questions was whether their organisation had adopted the alternate work modality during a pandemic. Of 302 respondents, 32 responded with No, and 270 indicated the development sector adopted the digital working mode, mitigating the Pandemic's effects on its operation and staff.

**Table 1:** Demographics.

Items	N	Percentage %
Male	190	62.9%
Female	99	32.8%
Prefer not to say	13	4.3%
SSC/O – level	2	0.7%
HSSC/A – level	7	2.3%
BA/BSc/BS	84	27.8%
MA/MSc/MS	197	65.2%
PhD	12	4.0%
No	32	10.6%
Yes	270	89.4%
1–5 years of experience	47	15.6%
6–10 years of experience	61	20.2%
11–15 years of experience	88	29.1%
16–20 years of experience	54	17.9%
20 and above, years of experience	38	12.6%

***Notes***: N = 302.

### 4.2 Model fit indices

CMIN stands for the Chi-square value and indicates if the relationship between the sample data and the model is a good fit. It is the fit goodness test if the model has deviated from the one that sits nicely on the data [85]. In Table 2, CMIN (chi-square) is 484.687, degree of freedom (DF) is 265, P is .000, and CMIN/DF is 1.82, between the threshold stated.

**Table 2:** CMIN.

Model	NPAR	CMIN	DF	P	CMIN/DF
Default model	60	484.687	265	.000	1.829
Saturated model	325	.000	0		
Independence model	25	3962.274	300	.000	13.208

With a sample size of more than 200, this could be disregarded if other indices indicate an acceptable model [86], While as per [85] if the value is ≤ 3, it is an acceptable fit. *P*-value denotes the significance level; traditionally, if p ≤ .05, we reject the null of an exact-fitting model. The chi-square result shows that the null of an exact-fitting model (DF 265, CMIN 484.687, P < .05) is to be rejected.

### 4.3 Baseline comparisons

The Normed fit index (NFI), Relative fit index (RFI), Incremental fit index (IFI), Tucker-Lewis Index, and Comparative fit index (CFI) are comparative fit indices that compare the model fit against the null/independence model [87,88]. They account for model complexity in their RFI, IFI, NNFI, and CFI, accounting for model complexity in calculations (Table 3).

**Table 3:** Baseline comparison.

Model	NFI Delta1	RFI rho1	IFI Delta2	TLI rho2	CFI
Default model	.878	.862	.941	.932	.940
Saturated model	1.000		1.000		1.000
Independence model	.000	.000	.000	.000	.000

The CFI represents the discrepancy ratio between the observed and independent models. It shows how the experimental model is better than the independent model. Indices range between 0 and 1, with values ≥ 0.90, are treated as indicative of an acceptable fitting model, while values ≥ 0.95 are considered to be a ‘superior fit’ [88]. As per [86], CFI is not affected by sample size. The current value of 0.940 is in an acceptable range. TLI and CFI comparative indices are reported commonly. TLI (0.932) and CFI (0.940) fit in the excellent range.

### 4.4 RMSEA

RMSEA is a Root-mean-square error of approximation (RMSEA) that could be said as an ‘absolute fit index’, with 0 indicating the ‘best fit and values > 0 suggesting a worse fit [85]. Values of 0.05 or below indicate a close-fitting model, while values between/up to .08 or 0.10 are deemed acceptable [89,90].

In Table 4, the RMSEA = 0.052 indicates that the model is a close fit to the data. Another way to assess the model fit is PCLOSE. The test helps determine a model's fit based on the RMSEA. Suppose the value of RMSEA is ≤ 0.05, representing a close-fitting model, then P > .05 represents the supporting null hypothesis of close model fit [85]. In our current analysis, PCLOSE is .283, suggesting the null hypothesis of close fit – it supports our model. The HI 90 in the table (upper bound) is > 0.05. If it were <0.05, then it would have been a close model fit, while if it was >0.10 (the threshold for ‘poor fit), then we have weaker evidence in support of a well-fitting model (Kline, 1998). The lower bound (LO) of the interval is < 0.05, that is 0.045, and the upper bound is < 0.10 and >0.05, that is 0.060, providing support that model is an acceptable fit to the data. The lower bound of our output shows a close-fitting model, whereas the upper bound suggests a poor-fitting model; therefore, we resolved the apparent contradiction by stating that the model is ‘just as consistent with the close-fit hypothesis as it is with the poor-fit hypothesis’. The summary of the model fit is in Table 5.

**Table 4:** RMSEA.

Model	RMSEA	LO 90	HI 90	PCLOSE
Default model	.052	.045	.060	.283
Independence model	.201	.196	.207	.000

**Table 5:** Model fit indices.

Measure	Estimate	Threshold	Interpretation
CMIN	484.687	–	–
DF	265.000	–	–
CMIN/DF	1.829	Between 1 and 3	Excellent
CFI	0.940	>0.95	Acceptable
SRMR	0.059	<0.08	Excellent
RMSEA	0.052	<0.06	Excellent
PClose	0.283	>0.05	Excellent

**Notes**: *Hu and Bentler (1999) “Cutoff Criteria for fit Indexes in Covariance Structure Analysis: Conventional Criteria Versus New Alternatives*.

### 4.5 Reliability analysis

The Cronbach's Alpha test is used to measure the reliability of the data that the scale measures. Table 6 shows that Cronbach's alpha for all items is > 0.70. If there are less than ten items on the scale, it is not easy to get a high alpha [91]. So, in that case, the value should be > 0.5. Here, we have values greater > than 0.70 that show high reliability [92] has given us the best parameter to describe reliability:”>0.9 = Excellent, >0.8 = Good, >0.7 = Acceptable, >0.6 = Questionable, >0.5 = Poor, and if the values are <0.5, then it is unacceptable.”

**Table 6:** Reliability statistics.

	Cronbach's Alpha	N of Items
Digital Communications	.845	6
Ease of Use	.790	5
Top Management Support	.908	8
Project Performance	.879	6

In Table 6, the digital communication Cronbach's alpha is .845, and for a project performance is .879. This shows a good correlation between the items and indicates that the data is reliable. For Ease of Use, the ɑ is 0.790, showing that reliability is acceptable, while Top Management supports the ɑ is 0.908, indicating excellent reliability.

### 4.6 Descriptives statistics

The descriptive of the study shows that the data taken into consideration is 302. The variable values of the survey minimum are one (1), and the maximum is 5. The range for all the variables is the same. The mean for the different variables is around two, showing that data is distributed around 2. In Table 7, the standard deviation for digital communication (DC) and top management support (TMS) is 0.9, while for ease of use (EU) and project performance (PP) is 0.8, which shows that data is within the range of 1hSD (standard deviation) and indicate that the sample size is not a large one. Also, it shows that the data is close to the mean value (LabCE, n.d.).

**Table 7:** Descriptive statistics.

		DC	EU	TMS	PP
N	Valid	302	302	302	302
	Missing	0	0	0	0
Mean		2.2169	2.3987	2.1908	2.4111
Std. Deviation		.94445	.89902	.99915	.86251
Skewness		.592	.812	.379	.582
Std. Error of Skewness		.140	.140	.140	.140
Kurtosis		.019	.720	−.875	.655
Std. Error of Kurtosis		.280	.280	.280	.280
Minimum		1.00	1.00	1.00	1.00
Maximum		5.00	5.00	5.00	5.00

***Notes:****Digital Communication (DC), Ease of Use (EU), Top Management Support (TMS), Project Performance (PP*).

Kurtosis for digital communication is .01, ease of use is 0.72, top management support is −0.87, and project performance is .655. The data shows that it is within the range of + and – 1. This indicates that the kurtosis for all variables is within the normal range, so the distribution is normal. Kurtosis measures the heaviness of the data; either it will be a light or heavily weighted tail. Our data output shows that the data has a normal tail [93] Skewness is to measure the symmetry, in other words, to check if the data lacks balance. The skewness for data is within the range of + and – 1, making it normal. Had the skewness been more significant than +1, then the data distribution would have been skewed to the right, while if the value is −1, then the data would be skewed to the left [93].

### 4.7 Correlation

The next step in the analysis is the correlation—a technique used to show how variables relate to each other and the strength of their relation. In our study, the correlation used is Pearson's r. The r ranges between −1.0 and + 1.0. Correlation 0 means no relationship. The closer the value to −1 or +1, the more substantial relationship between the variables, while – and + with correlation coefficient indicates the increase or decrease of the correlation in the relationship. Table 8 shows that digital communication positively correlates with ease of use, top management support and project performance. There are guidelines to assess the strength of the correlation below [94] : .1 < |r | < 0.3 - weak correlation, 0.3 < |r | < 0.5- moderate correlation and 5 < |r | is a strong correlation.

**Table 8:** Correlation matrix.

		1	2	3	4
Correlation	DC	–			
	EU	.387**			
	TMS	.461**	.466**		
	PP	.384**	.368**	.432**	

**Notes**: N = 302, **p < .01, Digital Communication (DC), Ease of Use (EU), Top Management Support (TMS), Project Performance (PP).

Per guidelines, the DC has a weak correlation with EU and PP and a moderate correlation with TMS. EU has a moderate correlation with TMS and a weak correlation with PP. TMS has a reasonable correlation with PP. So the takeaways are 1) digital communication, ease of use, top management support, and project performance are positively correlated. If one variable increases, the rest will increase too. 2) As the variables are positively correlated, this indicates that they have a linear relationship as variables increase together. 3) The overall strength of the correlation among the variables is significantly moderate.

### 4.8 Regression/moderation

The next step in the analysis is to run the regression analysis. The process is initiated with linear regression, which is run to analyse the impact of digital communication on project performance in the absence of moderators. The analysis values of the study are in Table 9.

**Table 9:** Model summary^b^.

Model		R	R Square	Adjusted R Square	Std. Error of the Estimate
		.375^a^	.141	.138	.73399

a Predictors: (Constant), DC.

b Dependent Variable: PP.

The R-value is a regression in Table 9 representing the correlation between digital communication and project performance. The R-square value shows that 0.141 = 14.1% of the variability in project performance has accounted for digital communication. Less than 15% of the impact is accounted for by digital communication; it is a meaningful predictor but not huge. The adjusted R square is an exciting value with a sample size of 302. The SPSS has adjusted the R square to 0.138 = 13.8% of variability <15%. As the data deviate from the model's actual mean, the standard error would not be accurate. In the table, the standard error is valid for the model with a sample size of 302. The analysis shows that digital communication does have an impact on performance management. The effect is termed as significant as in the present working condition. When the usual business has shifted to alternate modes of working, a 13.8% impact is also significant to help achieve the project goals.

To further analyse digital communication and project performance in the presence of moderators, the study adopted Hayes processes macro model 2 [95]. In model 2, we have two moderating variables, so we run the regression along with the covariates of Qualification (Q), Gender (G), Age (A), Education (E), and Working Status (WS.)

The R in Table 10 represents the correlation between the dependent variable – Performance management, and the independent variable - Digital Communication. The value of correlation is 0.6088 > 0.4, which is good. R^2^ is 0.3707, indicating that the change occurred is 0.37 (37%) on the project performance due to the moderation effect. To check if the variation created is significant or not, we take p = .000, indicating that it is substantial as the value is < 0.05. It also shows that the model has run correctly and the relationship between IV and DV is meaningful.

**Table 10:** Model summary.

	R	R^2^	P
	.6088	.3707	.0000

**Notes**: Correlation (R), Moderation effect (R^2^).

In Table 11, the Interaction 1 value is 0.0026, which is < 0.05 making it significant, while the Interaction 2 value is 0.3971, which is > 0.05, making it insignificant. The R^2-^change for interaction terms 1 and 2 indicates the variation in a dependent variable – project performance. Interaction effect 1 accounts for 0.0199, and Interaction 2 accounts for 0.0016 variation. While the impact of both interaction terms jointly accounts for a 0.0220 variation on the dependent variable.

**Table 11:** Model details.

	Β	R^2 Change^		P
DC	.0399			
EU	.2364			
TMS	.2929			
Interaction Term 1 (DC*EU)	.1621	.0199		.0026
Interaction Term 2 (DC*TMS)	−.0412	.0016		.3971
Interaction 1 & 2		0.220		0.006
Q	−.0206			.0182
G	.0168			.1258
A	.0086			.2836
E	.0053			.3107
WS	−.0274			.0958

***Notes:****N = 302 Digital Communication (DC), Ease of Use (EU), Top Management Support (TMS), Project Performance (PP), Qualification (Q), Gender (G), Age (A), Education (E), Working Status (WS.)*.

The coefficient value of interaction term 2 is −0.0412, which indicates that one unit shift increase in independent variables (digital communication and top management support) jointly causes a decrease in the dependent variable – project performance. Also, we see that all three independent variables have a positive relationship,i.e. one unit shift increase in independent variables leads to an increase in the dependent variable. However, when an independent variable works with one moderator, it puts a collective impact on DV while other variables in the model are constant. Hence, this confirms that digital communication moderates the effects of both moderators on the project performance and the moderation effect. The moderation effect of top management support is insignificant, while ease of use is significant.

It is also to note that the impact of the one covariate – qualification has a significance of < .05, indicating that the covariate impacts the model. In coefficient, the effect is negative (−0.0206). The other covariate working status also hurts the model with a negative result (−0.0274); nevertheless, the significance is > 0.05, making it insignificant. While other covariates have a positive relationship with the model, they are insignificant regarding p-value, i.e. >.05.

Table 12 shows that the conditional effects of digital communication at the different levels of both moderators on the model are decreasing from −0.0540 to 0.1338. The slopes test at -1SD, Mean,+1SD is negative to positive. The significance value gradually goes from insignificant (0.4837) to significant (0.0213). It reflects how a decrease in the collective impact of moderators enhances the model's significance. This impact indicates an inverse relationship between the combined effects of moderators with the IV and DV. A summary of the hypothesis is in Table 13.

**Table 12:** Conditional effects of the focal predictor at values of the moderator(s).

EU	TMS	Effect	Se	T	P	LLCI	ULCI
−.8193	−.9425	−.0540	.0770	−.7013	.4837	−.2055	.0975
−.8193	.0000	−.0929	.0700	−1.3259	.1859	−.2307	.0450
−.8193	.9245	−.1317	.0899	−1.4652	.1440	−.3087	.0452
.0000	−.9245	.0788	.0779	1.0117	.3125	−.0745	.2321
.0000	.0000	.0399	.0532	.7505	.4535	−.0648	.1446
.0000	.9245	.0011	.0616	.0171	.9863	−.1202	.1223
.8193	−.9245	.2116	.1002	2.1117	.0356	.0144	.4088
.8193	.0000	.1727	.0677	2.5495	.0113	.0394	.3060
.8193	.9245	.1338	.0578	2.3157	.0213	.0201	.2476

**Table 13:** Summary of hypotheses testing.

No	Hypothesis Statement	Hypotheses Testing Result at 95? Confidence level	Results
H1	Digital communication tools significantly impact project performance	P = .000 < .05, Null hypothesis not rejected	Do not reject
H2	Top management moderates the relationship between digital communication tools and project performance	P = .6158 > 0.05, Null hypothesis rejected	Rejected
H3	Ease of use significantly moderates the relationship between digital communication tools and project performance.	P = .0022 < .05, Null hypothesis not rejected	Do not reject

## 5 Discussion

Organisations around the globe dealt with the Pandemic by switching to digital workspace; in Pakistan, organisations hesitantly adopted telecommuting. The new normal opened a further discussion among the researchers to work on the effects of virtual workplaces and their impact on organisations, keeping no other work choices constant. The study's finding shows that the two moderators (top management support & ease of use of digital communication tools) impact the relationship between the dependent variable – project performance and the independent variable – Digital communication.

While analysing the regression, we used the covariates to view their impact on the model. The covariate's qualification and work status negatively affect the model. The qualification has a high significance, while the work status is insignificant. For analysing the covariates, the demographic table is analyzed further. The findings are that 65.2% of the respondents hold a master's degree, and the majority of the respondent's responses could be making qualification inversely proportional to the project performance. So, the researcher understands that highly qualified respondents were unhappy with the project's performance during the lockdown. To explain this phenomenon, low satisfaction with the project performance is due to its Management [[96], [97], [98], [99]]. In general, respondents seem satisfied; however, their expectations are high. It resonates with the study conducted in Portugal, where seasoned professionals were practising online but were dissatisfied with their performance. The high-qualified workforce believed that organisations could take better measures but did not take the initiative. The alternative reasoning is that these people believed in person working more than telecommuting, which was impossible due to health safety and government restrictions.

To navigate through the turbulent time, the organisations, despite Covid-19, have maintained their performance goals and, with minor fine-tuning, tried to keep the shareholder's and employees' interests aligned [100]. This strategy has fired back in the case of employees. The working arrangement has burdened employees with the fear of not meeting the performance goals leading to downsizing. They have to juggle both professional and personal life, including the schools that also switched to digital tools, making it complicated for female participants [101]. A study on the general public has explained this psychological pressure and the impact of covid-19 on people in Bangladesh [102].

Ease of use is directly proportional to project performance - a positive relationship. The more user-friendly the communication tools, the more they will help achieve the performance goals. It explains the significant p-value <.05, but adopting ICT tools was cumbersome in the initial days of covid-19 and required attention and knowledge [19] 29.1% of study participants have 11–15 years of experience, whereas 20.2% are 6–10 years. People with more and moderate experiences continue to work online, although dissatisfaction with their performance reflects by the covariate qualification's significant negative impact on the model. So, it is safe to say that despite dissatisfaction, both of them continued to work regardless of positive or negative attitudes.

The second moderator's top management support findings are fascinating. To our surprise, interaction term 2 is inversely proportional – negative relationship to the project performance. Generally, most of the organisation's strategic decisions directly connect to top management support. However, the Pandemic was a global health issue; strict government measures compelled the organisation/projects to adopt the digital transformation making the top management support insignificant.

The difference between blue and white-collar jobs has affected the research. Blue-collar jobs must be at the site; white-collar jobs are usually in the office, and who could take advantage of flexible/virtual working? A study showed that around 20% of organisations have temporarily laid-off employees in Pakistan during a pandemic. 16–17% were temporarily laid-off without pay, and 7–8% were permanently laid-off without compensation [103]. The pressure exerted and the fear of being jobless during the challenging time is another critical view that leads to dissatisfaction with the performance during the lockdown.

One of the other factors that may have affected the performance is not so robust or uniform internet connectivity across the city. People are more comfortable working in person in an office environment. The new working arrangement has made them feel burdened with constant calls and emails with no off from work. People working can say no more “No” to any work put in their laps. They must do it, while this may involve coordination and interaction outside the organisation, making it more difficult due to the different working arrangements of other organisations. Even people within organisations are unfamiliar with working-from-home arrangements, so no valid policies or guidelines are provided or abide by all. This put extra pressure on the study's subjects and made them available to respond 24/7 (per their perception) [101]. Due to working from home, the lines between personal and professional life have blurred. In Pakistan, employers considered the uncommon concept of working from home a luxury, and many large organisations used to discourage it.

The unstable internet connection could also be called digital inequality. Digital inequality is due to people's insecurity about using digital tools, lacking skills for using the internet, e.g. digital illiteracy, or the temporary behaviour change due to the Pandemic. During the lockdown, people confined to homes relied on the internet to work and stay connected with family and work. The changed pattern from covid-19 and staying at home will last after the Pandemic? Will people continue to use the changed pattern of behaviour? Will they still work from home if they have the option to go back to the office? As for now, these questions can not be answered as the change's motivation and continuity are different. The pandemic has led people to new possibilities for using various digital communication tools. However, digitalisation has left people with a changed behaviour making it fundamental in everyday life.

In the survey, questions were asked if the top management communicated with the staff member using the organisation's different communication tools. The result showed that leaders in the organisation during the demanding phase in 2020 were understanding and communicated from time to time through emails and MS teams to keep morale high.

## 6 Theoretical and practical contribution

The current research has contributed to our understanding of digital tools in the changing working environment. Its socio-psychological impact on employees' professional lives and organisations’ responsibilities in the crisis context.

Covid-19 has brought about an irreversible shift to the people's livelihood and organisational operations. It has opened new technology doors and ascertained the need for new skills and survival of the fittest in the changing environment created by the Pandemic.

The study identifies the unintended pressure on employees working in the development sector of Islamabad, Pakistan. The pressure ranged from psychological impact to adherence to the organisational policies amid the covid-19. The study highlights the organisation's mechanism to understand its weakness in going through the turbulent water without hitting the underwater stones. It also confronts the employees that digitalisation of the workplace is the reality. The earlier they accepted, the more they could safely sail in the turbulent water.

## 7 Conclusion & recommendations

The study has brought grey areas to the surface during the Pandemic. It has contributed to our understanding of digital tools in the changing work environment and their socio-psychological impact on people's lives and organisations' responsibilities within the context of a pandemic. Researchers believe that health and economic crises might follow psychological emergencies during a study. People are affected mentally during confinement to homes regardless of the lockdown intensity [104].

The study's takeaway is that digital communication tools are vital in meeting performance goals within the new normal. The study has concluded different behaviour patterns of employees toward digital communication tools. The transformation from traditional to digital tools for working and communicating has pressured the professionals working in the humanitarian sector in Islamabad. The pressure ranged from psychological impact – to job security to adherence to the organisational policy amid the Pandemic. The overnight transformation has left the people and organisations in disarray. The inadequate preparedness to face the medical emergency has exposed the loopholes in the organisation's business continuity plan. At the same time, absence/limited training has led to difficulties in using communication tools, divulging the employees to inevitable change.

With the strict rule of law, communication between employees and employers was developed, creating a positive relationship: the restrictions-imposed help to understand each other (employee and supervisors) and expectations to some extent. Higher qualified employees have an inversely proportional connection with project performance. During a pandemic, the importance of user-friendly communication tools in the workplace has underscored overcoming work challenges.

Specific recommendations both for organisations and working professionals resulted from the discussion. The projects must devise an alternate working modality policy and business continuity plan. Conduct training for their workforce to introduce and implement them. Due to the changed behaviour pattern of the masses during the lockdown, there is a better scope for internet service providers to benefit from it. The projects should promote two-way communication, and people should focus on getting accustomed to the new technology.

A fair share of limitations is the study conducted in the capital city of Pakistan. Due to the absence of population size data in the Islamabad development sector, the Cochran formula calculates the sample size.

## Production notes

### Author contribution statement

Kainat Afridi: Conceived and designed the experiments; Performed the experiments; Analyzed and interpreted the data; Contributed reagents, materials, analysis tools or data; Wrote the paper.

Jamshid Ali Turi: Conceived and designed the experiments; Performed the experiments; Contributed reagents, materials, analysis tools or data.

Barirah Zaufishan: Conceived and designed the experiments; Analyzed and interpreted the data; Contributed reagents, materials, analysis tools or data.

JOANNA Rosak-Szyrocka: Conceived and designed the experiments; Analyzed and interpreted the data; Wrote the paper.

### Data availability statement

No data was used for the research described in the article.

### Additional information

No additional information is available for this paper.

## Declaration of competing interest

The authors declare that they have no known competing financial interests or personal relationships that could have appeared to influence the work reported in this paper.
