# Supplementary material for: Impact of digital communications on project efficiency through ease of use and top management support
Source: Heliyon. 2023 Jul 5;9(7):e17941. doi: 10.1016/j.heliyon.2023.e17941 (PMC10372203; doi:10.1016/j.heliyon.2023.e17941)
Supplement: Multimedia component 1 [file mmc1.docx]

Click or tap here to enter text.

**9. Appendices;**

| **Impact of digital communications on PP through ease of use and top management support** | | | | | | | | | | | | | |
| --- | --- | --- | --- | --- | --- | --- | --- | --- | --- | --- | --- | --- | --- |
| * Required | | | | | | | | | | | | | |
| About You   1. Are You. * (Mark only one ) | | | | | | | | | | | | | |
|  | Male | | | | | | | | | | | |  |
|  | Female | | | | | | | | | | | |  |
|  | Prefer not to say | | | | | | | | | | | |  |
| 1. Please indicate the age bracket you are in: ***** (Mark only one ) | | | | | | | | | | | | | |
|  | 20-25 | | |  | | | 26-30 | | | | | |  |
|  | 31-35 | | |  | | | 36-40 | | | | | |  |
|  | 41-45 | | |  | | | 46-50 | | | | | |  |
|  | 51-55 | | |  | | | 56-60 | | | | | |  |
|  | 61 and above | | |  | | | Prefer not to say | | | | | |  |
|  |  | | |  | | |  | | |  | |  |  |
|  |  | | |  | | |  | | |  | |  |  |
| 1. What is your highest Qualification? ***** (Mark only one ) | | | | | | | | | | | | | |
|  | SSC / O - level | | |  | | | HSSC / A - level | | | | | |  |
|  | BA / BSc / BS | | |  | | | MA / MSc / MS | | | | | |  |
|  | PhD | | |  | | | Other: | | | | | |  |
|  |  | | |  | | |  | | |  | |  |  |
| 1. Please indicate your year’s experience: ***** (Mark only one ) | | | | | | | | | | | | | |
|  | 1-5 | | |  | | | 6-10 | | | | | |  |
|  | 11-15 | | |  | | | 16-20 | | | | | |  |
|  | 20 and above | | |  | | | Prefer not to say | | | | | |  |
|  |  | | |  | | |  | | |  | |  |  |
| 1. Name of Organization you are working for: ***_____________________________** | | | | | | | | | | | | | |
| 1. Are you working on any project in your organisation? * (Mark only one ) | | | | | | | | | | | | | |
|  | Yes | |  | |  | No | | | | | |  |  |
|  |  | |  | |  |  | | | | | |  |  |
| 1. Title of the Project you are working on: *** ________________________________** | | | | | | | | | | | | | |
| **Digital Communications** | | | | | | | | | | | | | |
|  |  | | |  | | |  | | |  | |  |  |
| 1. Did your organisation opt for the alternate work modalities (e.g. work from home) during the lockdown in Covid-19?* (Mark only one ) | | | | | | | | | | | | | |
|  | Yes | | |  | | | No | | | | |  |  |
|  |  | | |  | | | |  | | | |  |  |
| 1. If yes then please state the work mode. *** ________________________________** | | | | | | | | | | | | | |
| 1. Did your Organization officially recommend/approved an online communication tool? (Mark only one ) | | | | | | | | | | | | | |
|  | Yes | | |  | | No | | |  | | |  |  |
| 1. If yes, which online communication tool is recommended by your organisation?* **(**Please select all that apply) | | | | | | | | | | | | | |
|  | Skype | | |  | | | Zoom | | | | | |  |
|  | MS Teams | | |  | | | Google Hangout | | | | | |  |
|  | WebEx | | |  | | | Face Time | | | | | |  |
|  | WhtsApp | | |  | | | Other: | | | | | |  |
|  |  | | |  | | |  | | |  | |  |  |
| 1. Did you face any difficulty while using the online communication tools during alternate work modalities ( e.g. work from home)?* (Mark only one ) | | | | | | | | | | | | | |
|  | Yes | | |  | | | No | | | |  |  |  |
|  |  | | |  | | |  | | | |  |  |  |
| 1. Refer to the question above: If yes, then please state what kind of difficulty you have encountered while using digital communication tools?* | | | | | | | | | | | | | |
|  | | | | | | | | | | | | | |
| 1. Were you using the digital/online Communication tools before the pandemic and lockdown ? (Mark only one ) | | | | | | | | | | | | | |
|  | Yes | | |  | | | No | | | |  |  |  |
|  | |  | |  | | |  | | |  | |  |  |
| Below are the questions that are used to collect the data from the field with a five Likert scale to respond to the survey questions. | | | | | | | | | | | | | |
| **Digital Communication** | | | | | | | | | | | | | |
| - During the lockdown without digital communica,tion my job would have been difficult to perform. | | | | | | | | | | | | | |
| - I get greater control over my work by using digital communication tools/mediums | | | | | | | | | | | | | |
| - Digital/online Communication tools have proved to be quite useful for professional work during panda emic | | | | | | | | | | | | | |
| - Digital Communication tools address all my job-related needs | | | | | | | | | | | | | |
| - Digital Communication tools have helped me in efficiently complete the tasks of the project during the lockdown | | | | | | | | | | | | | |
| - Digital Communication tools have enhanced my work performance on the project | | | | | | | | | | | | | |
| - Digital Communication tools have improved the quality of my work | | | | | | | | | | | | | |
| - The use of digital Communication tools has increased my productivity level during the alternate work modalities ( e.g. work from home) | | | | | | | | | | | | | |
| **Ease Of Use** | | | | | | | | | | | | | |
| - The digital/online Communication tools are easy to use. | | | | | | | | | | | | | |
| - I have learned to use digital/online communication tools during the alternate work modalities | | | | | | | | | | | | | |
| - I get confused while using digital/online communication tools | | | | | | | | | | | | | |
| - While using digital/online communication, I often make errors | | | | | | | | | | | | | |
| - Using digital/online communication tools, I need to consult user manual | | | | | | | | | | | | | |
| - Using digital/online communications tools required a lot of mental effort | | | | | | | | | | | | | |
| - I find it easy to interact with colleagues by using digital/online communications tools | | | | | | | | | | | | | |
| - The digital/online communications tools are rigid and inflexible to interact | | | | | | | | | | | | | |
| - The use of digital/online communications makes the interaction with colleagues and supervisors transparent and understandable | | | | | | | | | | | | | |
| - The use of digital/online communications provides adequate information to the relevant people/stakeholder | | | | | | | | | | | | | |
| - Digital/online communications help information distribution easily and effectively | | | | | | | | | | | | | |
| **Top Management Support** | | | | | | | | | | | | | |
| - Top management understands the importance of communication tools during the lockdown | | | | | | | | | | | | | |
| - Top management supports the use of online communication tools for accomplishing the project goals | | | | | | | | | | | | | |
| - Top management see online mediums as strategically crucial for the organisational goals unaffected during the lockdown | | | | | | | | | | | | | |
| - Top management understands the transformation to online medium as an opportunity to survive during the pandemic | | | | | | | | | | | | | |
| - Top management provided the resources to facilitate the transformation from traditional to online communication | | | | | | | | | | | | | |
| - Top management frequently communicated with internal and external stakeholders using online communication tools during a pandemic | | | | | | | | | | | | | |
| - Top management uses and keeps the pressure on teamwork by using online mediums to survive during a pandemic | | | | | | | | | | | | | |
| - Top management provided the training to facilitate the transformation from traditional to online communication | | | | | | | | | | | | | |
| - Top management provided sufficient resources to support the digital adaptation of work in the organisation during a pandemic | | | | | | | | | | | | | |
| - Top management regularly communicated with the project team members to ensure digital communication adaptation | | | | | | | | | | | | | |
| - Top management tailored communication to digital platforms to emphasise the significance of project | | | | | | | | | | | | | |
| - Top management encouraged frequent communication to discuss problems faced by the project team members by using digital communication tools | | | | | | | | | | | | | |
| - Top management ensured an effective transition to digital communication tools | | | | | | | | | | | | | |
| **Project Performance** | | | | | | | | | | | | | |
| All Project Assignments were followed as per the planned schedule during Covid | | | | | | | | | | | | | |
| - The schedule for each phase of the project was essentially the same as planned before the pandemic | | | | | | | | | | | | | |
| - Major project activities were completed on schedule during Covid | | | | | | | | | | | | | |
| - The project was delivered/will be delivered on schedule during Covid | | | | | | | | | | | | | |
| - The cost objectives were met in the project during Covid | | | | | | | | | | | | | |
| - The budget for each phase of the project was essentially the same as planned before the pandemic | | | | | | | | | | | | | |
| - The overall budget for the project remained essentially the same as planned during Covid | | | | | | | | | | | | | |
| - The project Deliverables produced were of high quality during Covid | | | | | | | | | | | | | |
| - The quality objectives were achieved for the project during Covid | | | | | | | | | | | | | |
| - The project was performed based on the client’s requirements during Covid | | | | | | | | | | | | | |
| - The project Owner was satisfied with the project’s deliverables and the project management process during Covid | | | | | | | | | | | | | |
| - The project team was satisfied with the project’s deliverables and the project management process during Covid | | | | | | | | | | | | | |
|  | | | | | | | | | | | | | |
